# Entry, egress and vertical transmission of SARS-CoV-2

**DOI:** 10.1093/jmcb/mjab013

**Published:** 2021-03-01

**Authors:** Hui Zhang, Hong Zhang

**Affiliations:** 1 National Laboratory of Biomacromolecules, CAS Center for Excellence in Biomacromolecules, Institute of Biophysics, Chinese Academy of Sciences, Beijing 100101, China; 2 College of Life Sciences, University of Chinese Academy of Sciences, Beijing 100049, China

**Keywords:** SARS-CoV-2, COVID-19, ACE2, TMPRSS2, placental transmission

## Abstract

The high infectivity and pathogenicity of severe acute respiratory syndrome coronavirus 2 (SARS-CoV-2) have caused the COVID-19 outbreak, one of the most devastating pandemics in more than a century. This pandemic has already left a trail of destruction, including enormous loss of life, a global economic slump, and widespread psychological damage. Despite assiduous world-wide endeavors, an effective cure for COVID-19 is still lacking. Surprisingly, infected neonates and children have relatively mild clinical manifestations and a much lower fatality rate than elderly adults. Recent studies have unambiguously demonstrated the vertical transmission of SARS-CoV-2 from infected pregnant women to fetuses, which creates yet another challenge for disease prevention. In this review, we will summarize the molecular mechanism for entry of SARS-CoV-2 into host cells, the basis for the failure of the lungs and other organs in severe acute cases, and the evidence for congenital transmission.

## Introduction

COVID-19 was first discovered in December 2019 in Wuhan, China, and then rapidly spread throughout the world. Its causative virus, SARS-CoV-2, is an enveloped, positive-sense, single-stranded RNA β-coronavirus. The SARS-CoV-2 genome, which comprises 29.9 kb, is coated with nucleocapsid (N) protein and enclosed in a lipid bilayer embedded with three membrane structural proteins, spike (S), envelope (E), and membrane (M). After the viral genome is released into the host cell, it is transcribed and translated to yield polyproteins that are further processed into 16 non-structural proteins (Nsps). Nsps constitute the replication and transcription complex and also interact with host factors to modulate the host immune response. Subgenomic mRNAs encode four structural proteins (S, E, M, and N proteins) and nine accessory factors. The accessory proteins also regulate viral–host interactions. Detailed molecular mechanisms for viral replication, transcription, and interaction with the host cells have been excellently reviewed recently (Hartenian et al., 2020). Despite the fact that SARS-CoV-2 exhibits 79.5% sequence identity with the SARS virus SARS-CoV and 96.2% identity with a bat coronavirus (Lu et al., 2020; Zhou et al., 2020), SARS-CoV-2 has much higher infectivity and has had a more profound global impact. In this review, we will discuss the possible molecular basis for the differential infectivity and damage of SARS-CoV-2 in adults and infants/children. Evidence for the vertical transmission of SARS-CoV-2 from the infected pregnant mother to the fetus will also be presented.

**Entry of SARS-CoV-2 into host cells**

To enter the cell, the surface-anchored S protein of SARS-CoV-2 binds to the angiotensin converting enzyme 2 (ACE2) receptor of target cells to trigger the initial attachment (Hoffmann et al., 2020; Zhou et al., 2020). The SARS-CoV-2 S protein is present as a homotrimer with each subunit composed of two domains, the receptor-binding S1 domain and the S2 domain that mediates membrane fusion. S protein is activated by proteolytic cleavage at the junction between the S1/S2 domains and then at a site within the S2 domain, called the S2' site, by the host cell surface serine protease TMPRSS2 (Hoffmann et al., 2020). After this activation process, which is known as priming, S1 dissociates, leading to a drastic structural change of the S2 domain to allow fusion of viral and cellular membranes. SARS-2-CoV also exploits endocytosis as an entry route (Figure 1). The virus-containing endosomes fuse with lysosomes, in which cathepsin proteases mediate cleavage of S proteins to trigger viral/host membrane fusion.

ACE2 and TMPRSS2 are also employed by the SARS virus SARS-CoV for entry into target cells. SARS-CoV-2 S protein displays 76% protein identity with that of SARS-CoV. The receptor-binding domain (RBD) of SARS-CoV-2 S protein has a 10-to-20-fold higher binding affinity to ACE2 than SARS-CoV S RBD (Hartenian et al., 2020). Moreover, the S1/S2 cleavage site of SARS-CoV-2 S contains several arginine residues, constituting motifs recognized by the cellular protease FURIN, which are absent in SARS-CoV S protein (Hoffmann et al., 2020; Shang et al., 2020). TMPRSS2, lysosomal cathepsins, and FURIN have all been implicated in the subsequent cleavage at the S2' site of S protein (Hartenian et al., 2020; Hoffmann et al., 2020; Shang et al., http://mc.manuscriptcentral.com/jmcb

2020). Pre-cleavage of SARS-CoV-2 S protein at the S1/S2 junction site by FURIN that non-covalently associates with virus particles when they are synthesized in host cells potentiates membrane fusion for virus entry into new target cells (Hartenian et al., 2020). An increased affinity of SARS-CoV-2 S protein for the ACE2 receptor and pre-cleavage of S protein by FURIN contribute to the higher infectivity of SARS-CoV-2 compared to SARS-CoV (Shang et al., 2020). Inhibiting ACE2 binding (e.g. with neutralizing antibodies), inactivating host proteases (e.g. by using the TMPRSS2 inhibitor camostat mesylate), and blocking S2-mediated membrane fusion have been explored as strategies for developing therapeutics (Hoffmann et al., 2020; Shang et al., 2020).

**Egress of SARS-CoV-2 from host cells**

The replication and transcription complexes (RTCs) of coronaviruses, such as MERS-CoV, murine hepatitis virus (MHV), and SARS-CoV, are anchored on double-membrane vesicles (DMVs) (Knoops et al., 2008; Snijder et al., 2020). SARS-CoV-2 infection also induces the formation of DMVs in host cells (Snijder et al., 2020; Miao et al., 2021), and the viral RNA products are stored in DMVs and trafficked from the interior to the cytosol for translation or viral assembly via double membrane-spanning pores (Wolff et al., 2020). During the egress stage of β-coronaviruses, new viral particles are assembled in the endoplasmic reticulum (ER) and/or ER–Golgi intermediate compartment (ERGIC) and subsequently released via a lysosomal exocytic pathway (Figure 1; Ghosh et al., 2020; Knoops et al., 2008; Snijder et al., 2020). The presence of SARS-CoV-2 virions in other types of vesicles in infected cells, such as double-membrane structures, smooth-walled vesicles, and autophagic or endomembrane compartments (Bradley et al., 2020; Pizzorno et al., 2020; Miao et al., 2021), suggests that the virions can be released via other trafficking routes.

Multiple surveillance mechanisms in host cells are activated to combat invading viruses. One such defense system is autophagy, a process involving the delivery of viruses or viral proteins in double-membrane autophagosomes to lysosomes for degradation. Autophagy also delivers viral nucleic acids and antigens to endolysosomal compartments for activation of innate and adaptive immune responses (Deretic et al., 2013; Dreus and Chisari, 2010). SARS-CoV-2 utilizes various strategies to evade autophagic destruction. Autophagy activity is blocked in SARS-CoV-2-infected cells. In cells infected with SARS-CoV-2 or expressing the viral protein ORF3a, the tethering complex HOPS is sequestrated on late endosomes, thereby blocking assembly of the SNARE complex that mediates fusion of autophagic structures with lysosomes (Miao et al., 2021). Other viral factors also block the progression of autophagic flux through other mechanisms such as causing the formation of small-size autophagosomes. The degradative capability of lysosomes is also impaired by ORF3a expression (Ghosh et al., 2020; Miao et al., 2021), which may facilitate the http://mc.manuscriptcentral.com/jmcb lysosome-mediated release of SARS-CoV-2. ORF3a from the SARS virus SARS-CoV fails to interact with HOPS or block autophagy (Miao et al., 2021). The unique function of SARS-CoV-2 ORF3a may contribute to the high infectivity and pathogenicity of SARS-CoV-2.

**Dynamic expression of ACE2 and TMPRSS2**

Organs with expression of ACE2 and TMPRSS2 are potentially susceptible to SARS-CoV-2 infection. Protein and/or mRNA levels of ACE2 and TMPRSS2 have been analyzed in various tissues at different ages. ACE2 is expressed in a wide spectrum of tissues, including the respiratory, cardiovascular, digestive, and urinary systems. The transcriptome data for *ACE2* and *TMPRSS2* at the single-cell level show that they are expressed in the lung tissue and subsegmental bronchial branches. Interestingly, *ACE2* is found to be strongly expressed in bronchial transient secretory cells (Lukassen et al., 2020). *FURIN* is also expressed in these tissues (Lukassen et al., 2020). By enhancing SARS-CoV-2 S protein priming after ACE2 binding, FURIN in the respiratory tract will render more cells permissive, and/or enhance the permissiveness of cells, to infection (Lukassen et al., 2020). ACE2 is expressed in the male and female reproductive system. TMPRSS2 is also expressed in spermatogonia and spermatids (Li et al., 2020). COVID-19 patients show white-matter injury. Consistent with this, the oligodendrocytes in the brain express both ACE2 and TMPRSS2 (Needham et al., 2020).

The transcriptomics data also indicate an age-dependent decrease in ACE2 expression in humans, which appears to be more significant in males than females (Chen et al., 2020). Levels of ACE2 expression are significantly decreased in type II diabetic patients (Chen et al., 2020). The age-dependent expression of ACE2 has also been characterized in rat lung. ACE2 protein is present in alveolar and bronchiolar epithelium and endothelium and smooth muscle cells of pulmonary vascular structures in rat lung tissues (Xie et al., 2006). ACE2 protein in rat lung is dramatically reduced with aging in both genders (Xie et al., 2006). ACE2 is also downregulated by inflammatory cytokines in various human and mouse tissues (Chen et al., 2020). Downregulation of ACE2 protein in mouse lung is also detected during SARS-CoV infection, treatment with the SARS-CoV S protein, and acid inhalation-induced lung injury (Imai et al., 2005; Kuba et al., 2005). SARS-CoV-2 infection may also decrease the ACE2 protein level.

**SARS-CoV-2 infection and cytokine storm**

Virus infection triggers the host innate immune response. In the early stages of virus infection, type I interferons (e.g. IFN-α and IFN-β) are important mediators of antiviral activity. They also stimulate the activation of immune cells (such as monocytes, macrophages, and natural killer cells), http://mc.manuscriptcentral.com/jmcb the initiation of the adaptive immune response, and the production of chemokines and cytokines such as interleukin-6 (IL-6) and tumor necrosis factor-α (TNF-α) (De Luca et al., 2020). In severe cases of COVID-19, induction of antiviral IFNs is compromised and there is also massive production of pro-inflammatory cytokines and chemokines such asIL-2, IL-6, IL-7, TNF-α, granulocyte colony stimulating factor (G-CSF), monocyte chemoattractant protein 1 (MCP-1), and macrophage inflammatory protein 1-a (MIP-1a), a phenomenon known as cytokine storm (De Luca et al., 2020). SARS-CoV-2 proteins have been shown to interact with host factors that restrict induction of type I IFNs, while stimulating pro-inflammatory cellular responses (Gordon et al., 2020; Hartenian et al., 2020). Disease manifestations in patients with severe respiratory failure due to SARS-CoV-2 infection are driven by systemic inflammation or cytokine storm, which recruits inflammatory cells such as neutrophils and cytotoxic T cells to induce severe lung tissue damage. Administration of IFNs and immunomodulatory therapies such as tocilizumab, a humanized monoclonal antibody against the IL-6 receptor, have been used for treatment of adult patients with severe COVID-19.

**ACE2 in the renin–angiotensin system**

The renin–angiotensin system maintains blood pressure homeostasis and fluid and salt balance through effects on the heart, blood vessels, and kidneys (Boehm and Nabel, 2002). Angiotensin II (Ang II), which is a peptide hormone and a potent blood vessel constrictor, binds to the G protein-coupled receptors, Ang II receptor type 1 (AT1R) and AT2R, to exert its effects. AT1R mediates the effects of Ang II on vasoconstriction and cardiac and vessel hypertrophy, while AT2R has opposing effects (Boehm and Nabel, 2002). The renin–angiotensin system is counterbalanced by ACE and ACE2. ACE cleaves the decapeptide Ang I into the octapeptide Ang II, while ACE2 reduces the levels of Ang II by cleaving a residue from Ang I to generate Ang 1–9 and a residue from Ang II to generate Ang 1–7, a blood-vessel dilator (Boehm and Nabel, 2002; Patel et al., 2017). Dysregulation of the renin–angiotensin system has a crucial role in modulating the severity of acute lung injury in mouse models. ACE2 and AT2R protect mice from severe acute lung injury induced by acid aspiration or sepsis (Imai et al., 2005). A decrease in ACE2 expression increases production of Ang II, causing increased pulmonary vascular permeability and loss of vascular homeostasis, subsequently exacerbating severe acute lung failure (Imai et al., 2005; Kuba et al., 2005). The severe lung failure and lethality associated with SARS-CoV-2 could be linked to the renin–angiotensin system, and levels of ACE2 expression may be associated with the severity of the lung pathogenesis. ACE2 expression shows a negative correlation with COVID-19 severity and fatality at a population level.

http://mc.manuscriptcentral.com/jmcb

## Symptoms of COVID-19 in newborns and children

Typical clinical symptoms of COVID-19 patients include fever, cough, diarrhea, fatigue, breathing difficulties, and pneumonia with characteristic ground-glass lung opacities (Dong et al., 2020). COVID-19 patients also exhibit neurologic manifestations such as dizziness, headache, impaired consciousness, acute cerebrovascular disease, taste impairment, smell impairment, and manifestations of skeletal muscular injury (Mao et al., 2020; Needham et al., 2020). Patients with severe infection according to their respiratory status are more likely to develop acute cerebrovascular disease, perturbed consciousness, and skeletal muscle injury (Mao et al., 2020). Deaths caused by SARS-CoV-2 infection are mainly seen in critical patients with preexisting comorbidities such as cardiovascular disease, diabetes, chronic respiratory disease, hypertension, and cancer.

Children with SARS-CoV-2 infection show milder clinical manifestations compared to adults (Sinha et al., 2020; Tung Ho et al., 2020). Common symptoms present in children during the illness include fever, cough, pharyngeal erythema, and features of pneumonia (De Luca et al., 2020; Lu et al., 2020). COVID-19 children may suffer from psychosocial impact caused by school closure, lack of outdoor activity, and other changes of routine habits, which can elicit various neuropsychiatric manifestations (Ghosh et al., 2020). More recently, accumulating evidence indicates that a few weeks after the onset of COVID-19, SARS-CoV-2 infection can cause the development of a hyperinflammatory syndrome resembling Kawasaki disease in older children (>5 years), which is named as multisystem inflammatory syndrome in children (MIS-C) (Godfred-Cato et al., 2020; Nakra et al., 2020; Panupattanapong and Brooks, 2020; Kabeerdoss et al., 2021). The clinical manifestations of MIS-C include fever, rash, gastrointestinal symptoms, shock, inflammation, cardiovascular complications, and positivity for SARS-CoV-2 (Godfred-Cato et al., 2020; Kabeerdoss et al., 2021). Thus, SARS-CoV-2 infection can trigger post-infectious immune dysregulation (Nakra et al., 2020; Kabeerdoss et al., 2021).

Zeng et al. (2020) reported a spectrum of illness in 3 out of 33 neonates who were born to mothers affected by COVID-19 and tested positive for SARS-CoV-2 through pharyngeal and anal swabs on day 2 after birth (Zeng. During the illness, the infected neonates showed shortness of breath, lethargy, vomiting, fever, respiratory distress syndrome, and pneumonia (Zeng et al., 2020). Bilateral gliosis of the deep white periventricular and subcortical matter was observed in a neonate 11 days after birth, possibly resulting from the vascular inflammation induced by SARS-CoV-2 infection (Vivanti et al., 2020).

Various hypotheses have been proposed to explain the milder COVID-19 illness in children. The higher level of ACE2 in children may lessen the disease pathogenesis, although it might cause a higher viral attack rate. Compared to elderly patients, children have a more effective immune system, such as a constitutionally higher number of lymphocytes, and greater thymus activity, which produces a favorable CD4/CD8 ratio. A higher incidence of other viruses in the lungs and upper airways of children may also limit the growth of SARS-CoV-2 by direct virus–virus competition or viral interaction. Moreover, elderly patients have a higher prevalence of preexisting comorbidities such as cancer, diabetes, and cardiovascular diseases. Future studies are needed to address the detailed mechanisms underlying the differential disease severity in populations with different ages.

**Vertical transmission from infected mothers to newborns**

COVID-19 is commonly transmitted from human to human through respiratory droplets, close contact, and aerosols (Chan et al., 2020). Recently discovered vertical transmission of SARS-CoV-2 from the infected pregnant woman to the fetus poses another risk to neonates.

*Expression of ACE2 in the placenta*

The placenta and decidua are the main maternal–fetal interfaces during pregnancy. The placenta plays a major role in preventing maternal–fetal transmission of pathogens. The major function of the placenta is maintained by trophoblast cells, including villous cytotrophoblasts (VCT), syncytiotrophoblasts (SCT), and extravillous trophoblasts (EVT). The SCT is the outer lining of the placental villi that directly contacts with maternal blood flowing into the intervillous space. A recent study using single-cell transcriptome data of early placenta (6–14 gestational weeks) revealed high expression of *ACE2* in the maternal–fetal interface including stromal cells and perivascular cells of the decidua and VCT and SCT in the placenta (Li et al., 2020). *TMPRSS2* is also found in VCT, SCT, and epithelial glandular cells (Epi) (Li et al., 2020). *ACE2* and *TMPRSS2* undergo a dynamic alteration in EVT, with low levels in the early placenta (8 weeks) and significantly increased levels at a later stage of pregnancy (24 weeks) (Li et al., 2020). Coexpression of *ACE2* and *TMPRSS2* in placenta and decidual cells suggests that the placenta is susceptible to SARS-CoV-2 infection, and the infection risk for the fetus may increase as the pregnancy progresses. Ang 1–7, produced by ACE2, facilitates vasodilation in the maternal–fetal circulation, which is favorable for virus spread.

ACE2 is also expressed in human fetal heart, liver, and lung (Li et al., 2020). High expression of ACE2 is found in airway epithelial cells and arterial endothelial cells of the human lung on post-natal day 1 (PND 1), which is distinct from the high expression in alveolar cells in adult lungs. A significant dynamic alteration of ACE2 and TMPRSS2 is also observed in murine lung before and after birth (Li et al., 2020). During late pregnancy, both ACE2 and TMPRSS2 are highly expressed in airway epithelial cells. Immediately after birth (PND 1–PND 3), ACE2 and TMPRSS2 are highly coexpressed in alveolar cells, ciliated cells, and club cells of murine lung. After PND 1–PND 3, http://mc.manuscriptcentral.com/jmcb

ACE2 and TMPRSS2 are expressed at a low level in the lung (Li et al., 2020). The wide spectrum of ACE2 expression suggests that newborns might be highly vulnerable to SARS-CoV-2 infection.

*The presence of virions in the placenta*

Several studies reported the detection of SARS-CoV-2 virus in the placenta, but not in fetal organs (Algarroba et al., 2020; Penfield et al., 2020; Valk et al., 2020). For example, in a 40-year-old pregnant Hispanic female with a severe COVID-19 infection, electron microscopy of the placenta at 28 gestational weeks identified virions invading SCT in placental villi and in the cell processes of fibroblasts of the placental villi. The infant was negative when tested for SARS-CoV-2 on PND 2 and PND 3 (Algarroba et al., 2020). Penfield et al. (2020) obtained placental or amnion/chorion swabs from 11 newly delivered mothers with COVID-19. Three of the 11 swabs tested positive for SARS-Cov-2, while none of infants tested positive for the virus on PND 1–PND 5.

*Vertical transmission*

Infection of neonates can occur via the transplacental or transcervical route or via environmental exposure. Congenital transmission was controversial at the beginning of the COVID-19 outbreak due to a lack of vigorous testing and prevention procedures. A neonatal congenital infection would be considered if the virus is detected in amniotic fluid collected prior to the rupture of membranes or in blood drawn early in life. Transplacental transmission may cause placental inflammation and neonatal viremia. The presence of viremia in only 1% of COVID-19 cases suggests that placental and fetal seeding might be rare (Ovali, 2020). Chen et al. (2020) reported that amniotic fluid and cord blood from six SARS-CoV-2-infected mothers were negative for the virus; throat swabs from all six neonates were also negative. As the pandemic continues and more thorough analysis has been performed, evidence for vertical transmission has emerged (Table 1). Zeng et al. (2020) found three cases of COVID-19 among 33 neonates born to infected mothers in Wuhan, China. Alzamora et al. (2020) also reported a case where the neonatal nasopharyngeal swab performed 16 h after delivery was positive for SARS-CoV-2. In these studies, strict infection control and prevention procedures were implemented during delivery. Zamaniyan et al. (2020) also identified an intrauterine infection case in a woman with severe COVID-19 disease who delivered at 32 gestational weeks. SARS-CoV-2 was identified in the amniotic fluid and the neonate also tested positive following nasal and throat swabs (Zamaniyan et al., 2020). Dong et al. (2020) reported that a neonate born to a mother with COVID-19 had elevated anti-SARS-CoV-2 IgM antibodies and abnormal cytokine levels 2 h after birth. IgM cannot be transferred across the placenta and can only be produced by the infected fetus (Dong et al., 2020). A recent study of a neonate born to a mother infected in the last http://mc.manuscriptcentral.com/jmcb trimester unambiguously demonstrated the transplacental transmission of SARS-CoV-2 from the mother to the fetus (Vivaniti et al., 2020). After systemic examination, the authors found that the virus was present in the placenta, amniotic fluid, and maternal and newborn blood. Signs of acute and chronic intervillous inflammation in the placenta also confirmed the virus infection (Vivaniti et al., 2020). The neonate exhibited neurological manifestations of infection (Vivaniti et al., 2020). Fenizia et al. (2020) also reported two cases of vertical transmission out of 31 mothers with SARS-CoV-2 infection. Viral RNA was detected in placental tissue as well as nasopharyngeal newborn swabs (Fenizia et al., 2020). Various factors such as the viral load, the time between illness onset and delivery, and the stage of pregnancy may regulate vertical transmission of SARS-CoV-2. Comprehensive analysis of pregnant women and neonates has been summarized in recent papers (Ovali, 2020; Trippella et al., 2020; Schwartz, 2020).

## Conclusions and perspective

The soaring number and mortality of COVID-19 patients, the staggering economic fallout, and the associated psychological disturbance create an urgent requirement for the scientific community to develop effective therapeutics and vaccines. Elucidating the influence of the virus–host interactions on the innate and adaptive immune responses is crucial to understand the pathogenesis of SARS-CoV-2. It is also important to understand how viral proteins interact with host factors to modulate various cellular functions for the benefit of viral proliferation. For example, how do viral proteins remodel the endomembrane systems to accommodate viral proliferation, assembly, and release? How are the various viral proteins, which are located at distinct subcellular sites, coordinately trafficked for virion packaging? Can SARS-CoV-2 enter the host cell independent of the ACE2 receptor?

The mechanisms that underlie the significantly lower incidence of severe illness and death in children remain poorly understood. Previous studies examined the mRNA levels of *ACE2* and *TMPRSS2*. Characterization of the spatiotemporal distribution of ACE2, TMPRSS2, and FURIN proteins in human tissues at the fetal and post-natal stages will provide insights into the potency of infant infection by SARS-CoV-2. Accurate diagnostic tools need be developed for children with non-specific symptoms. The severity of the pandemic demands combined global efforts for the future well-being of mankind.

## Acknowledgements

We are grateful to Dr Isabel Hanson for editing work.

http://mc.manuscriptcentral.com/jmcb

**Funding**

Work in Hong Zhang's laboratory was supported by grants to H.Z. from the National Natural Science Foundation of China (31421002, 31561143001, 31630048, and 31790403), Chinese Ministry of Science and Technology (2017YFA0503401), Beijing Municipal Science and Technology Committee (Z181100001318003), Strategic Priority Research Program of the Chinese Academy of Sciences (CAS) (grant XDB19000000), and Key Research Program of Frontier Sciences, CAS (grant QYZDY-SSW-SMC006).

**Conflict of interest**: none declared

## References

Algarroba, G.N., Rekawek, P., Vahanian, S.A., et al. (2020). Visualization of severe acute respiratory syndrome coronavirus 2 invading the human placenta using electron microscopy. Am. J. Obstet. Gynecol. *223*, 275-278.

Alzamora, M.C., Paredes, T., Caceres, D., et al. (2020). Severe COVID-19 during Pregnancy and Possible Vertical Transmission. Am. J. Perinatol. *37*, 861-865.

Boehm, M., and Nabel, E.G. (2002). Angiotensin-converting enzyme 2—a new cardiac regulator. New Engl. J. Med. *347*, 1795-1797.

Bradley, T.B., Maioli , H., Johnston, R., et al. (2020). Histopathology and ultrastructural findings of fatal COVID-19 infections in Washington State: a case series. Lancet *396,* 320-332.

Chan, J.F. Yuan, S., Kok, K.H., et al. (2020). A familial cluster of pneumonia associated with the 2019 novel coronavirus indicating person-to-person transmission: a study of a family cluster. Lancet *395*, 514-523.

Chen, H., Guo, J.,Wang, C, et al. (2020). Clinical characteristics and intrauterine vertical transmission potential of COVID-19 infection in nine pregnant women: a retrospective review of medical records. Lancet *395*, 809-815.

Chen, J.W., Jiang, Q.L., Xia, X., et al. (2020). Individual variation of the SARS-CoV-2 receptor ACE2 gene expression and regulation. Aging Cell *19,* e13168.

De Luca, C.D., Esposito, E., Cristiani, L., et al. (2020). Covid-19 in children: a brief overview after three months experience. Paediatr. Respir. Rev. *35,* 9-14.

Deretic, V., Saitoh, T., and Akira, S. (2013). Autophagy in infection, inflammation and immunity. Nat. Rev. Immunol. *13*, 722-737.

Dong, L., Tian, J., He, S., et al. (2020). Possible Vertical Transmission of SARS-CoV-2 From an Infected Mother to Her Newborn. JAMA *232*, 1846-1848.

Dong, Y., Mo, X., Hu, Y., et al. (2020). Epidemiology of COVID-19 Among Children in China. Pediatrics *145,* e20200702.

Dreux, M., and Chisari, F.V. (2010). Viruses and the autophagy machinery. Cell Cycle *9*, 1295-1307.

Fenizia, C., Biasin, M., Cetin, I., et al. (2020). Analysis of SARS-CoV-2 vertical transmission during pregnancy. Nat. Commun. *11*, 5128.

Ghosh, R., Dubey, M.J., Chatterjee, S., et al. (2020). Impact of COVID -19 on children: special focus on the http://mc.manuscriptcentral.com/jmcb psychosocial aspect. Minerva Pediatr. *72*, 226-235.

Ghosh, S., Dellibovi-Ragheb, T. A., Kerviel, A., et al. (2020). β-Coronaviruses Use Lysosomes for Egress Instead of the Biosynthetic Secretory Pathway. Cell *183*, 1520-1535.e14.

Godfred-Cato S., Bryant, B., Leung, J., et al. (2020). COVID-19-Associated Multisystem Inflammatory Syndrome in Children–United States, March–July 2020. MMWR Morb. Mortal. Wkly Rep. *69*, 1074-1080.

Gordon, D.E., Jang, G.M., Bouhaddou, M., et al. (2020). A SARS-CoV-2 protein interaction map reveals targets for drug repurposing. Nature *583*, 459-468.

Hartenian, E., Nandakumar, D., Lari, A., et al. (2020). The molecular virology of Coronaviruses. J. Biol. Chem. *295*, 12910-12934

Hoffmann, M., Kleine-Weber, H., Schroeder, S., et al. (2020). SARS-CoV-2 Cell Entry Depends on ACE2 and TMPRSS2 and Is Blocked by a Clinically Proven Protease Inhibitor. Cell *181*, 271-280.e8.

Imai, Y., Kuba, K., Rao, S., et al. (2005). Angiotensin-converting enzyme 2 protects from severe acute lung failure. Nature *436*, 112-116.

Kabeerdoss, J., Pilania, R.K., Karkhele, R., et al. (2021). Severe COVID-19, multisystem inflammatory syndrome in children, and Kawasaki disease: immunological mechanisms, clinical manifestations and management. Rheumatol Int. *41*, 19-23.

Knoops, K., Kikkert, M., van den Worm, S.H.E., et al. (2008). SARS-coronavirus replication is supported by a reticulovesicular network of modified endoplasmic reticulum. Plos. Biol. *6*, 1957-1974.

Kuba, K., Imai, Y., Rao, S., et al. (2005). A crucial role of angiotensin converting enzyme 2 (ACE2) in SARS coronavirus-induced lung injury. Nat. Med. *11*, 875-879.

Li, M.M., Chen, L., Zhang, J.X., et al. (2020). The SARS-CoV-2 receptor ACE2 expression of maternal-fetal interface and fetal organs by single-cell transcriptome study. Plos One *15*. e0230295.

Li, R., Yin, T., Fang, F., et al. (2020). Potential risks of SARS-CoV-2 infection on reproductive health. Reprod. Biomed. Online *41*, 89-95.

Lu, R., Zhao, X., Li, J., et al. (2020). Genomic characterisation and epidemiology of 2019 novel coronavirus: implications for virus origins and receptor binding. Lancet *395*, 565-574.

Lu, X., Zhang, L., Du, H., et al. (2020). SARS-CoV-2 Infection in Children. New Engl. J. Med. *382*, 1663-1665.

Lukassen, S., Chua, R.L., Trefzer, T., et al. (2020). SARS-CoV-2 receptor ACE2 and TMPRSS2 are primarily expressed in bronchial transient secretory cells. EMBO J. *39*, e105114.

Mao, L., Jin, H., Wang, M., et al. (2020). Neurologic Manifestations of Hospitalized Patients With Coronavirus Disease 2019 in Wuhan, China. JAMA Neurol. *77,* 683-690.

Miao, G., Zhao, H., Li, Y., et al. (2021). ORF3a of the COVID-19 virus SARS-CoV-2 blocks HOPS complex-mediated assembly of the SNARE complex required for autolysosome formation. Dev. Cell *56*, 427-442.

Nakra, N.A., Blumberg, D.A., Herrera-Guerra, A., et al. (2020). Multi-System Inflammatory Syndrome in Children (MIS-C) Following SARS-CoV-2 Infection: Review of Clinical Presentation, Hypothetical Pathogenesis, and Proposed Management. Children *7*, 69.

Needham, E.J., Chou, S.H.Y., Coles, A.J., et al. (2020). Neurological Implications of COVID-19 Infections. Neurocrit Care *32*, 667-671.

Ovali, F. (2020). SARS-CoV-2 Infection and the Newborn. Front Pediatr *8*, 294.

Patel, S., Rauf, A., Khan, H., et al. (2017). Renin-angiotensin-aldosterone (RAAS): the ubiquitous system for homeostasis and pathologies. Biomed Pharmacother. *94*, 317-325.

Penfield, C.A., Brubaker, S.G., Limaye, M.A., et al. (2020). Detection of SARS-COV-2 in placental and fetal membrane samples. Am. J. Obstet. Gynecol. MFM *2*, 100133.

Pizzorno, A., Padey, B., Julien, T., et al. (2020). Characterization and Treatment of SARS-CoV-2 in Nasal and Bronchial Human Airway Epithelia. Cell Rep. Med. *1*, 100059.

Schwartz, D.A. (2020). An Analysis of 38 Pregnant Women with COVID-19, Their Newborn Infants, and Maternal-Fetal Transmission of SARS-CoV-2: Maternal Coronavirus Infections and Pregnancy Outcomes. Arch. Pathol. Lab Med. doi: 10.5858/arpa.2020-0901-SA.

Shang, J., Wan, Y., Luo, C., et al. (2020). Cell entry mechanisms of SARS-CoV-2. Proc. Natl Acad. Sci. USA. *117*, 11727-11734.

Sinha, I.P., Harwood, R., Semple, M.G., et al. (2020). COVID-19 infection in children. Lancet Respir. Med. *8*, 446-447.

Panupattanapong, S., and Brooks, E.B. (2020). New spectrum of COVID-19 manifestations in children: Kawasaki-like syndrome and hyperinflammatory response. Cleve. Clin. J. Med. doi: 10.3949/ccjm.87a.ccc039.

Snijder, E.J., Limpens, R.W.A.L., de Wilde, A.H., et al. (2020). A unifying structural and functional model of the coronavirus replication organelle: Tracking down RNA synthesis. Plos Biol. *18*. e3000715.

Trippella, G., Ciarcia, M., Ferrari, M., et al. (2020). COVID-19 in Pregnant Women and Neonates: A Systematic Review of the Literature with Quality Assessment of the Studies. Pathogens *9*, 485.

Tung Ho, C.L., Oligbu, P., Ojubolamo, O., et al. (2020). Clinical Characteristics of Children with COVID-19. AIMS Public Health *7*, 258-273.

Valk, J.E., Chong, A.M., Uhlemann, A.C., et al. (2020). Detection of SARS-CoV-2 in placental but not fetal tissues in the second trimester. J. Perinatol. doi: 10.1038/s41372-020-00877-8.

Vivanti, A.J., Vauloup-Fellous, C., Prevot, S., et al. (2020). Transplacental transmission of SARS-CoV-2 infection. Nat. Commun. *11*, 3572.

Wolff, G., Limpens, R.W.A.L., Zevenhoven-Dobbe, J.C., et al. (2020). A molecular pore spans the double membrane of the coronavirus replication organelle. Science *369*, 1395-1398.

Xie, X.D., Chen, J.Z., Wang, X.X., et al. (2006). Age- and gender-related difference of ACE2 expression in rat lung. Life Sci. *78*, 2166-2171.

Zamaniyan, M., Ebadi, A., Aghajanpoor, S., et al. (2020). Preterm delivery in pregnant woman with critical COVID-19 pneumonia and vertical transmission. Prenat. Diagn. *40,* 1759-1761.

Zeng, L., Xia, S., Yuan, W., et al. (2020). Neonatal Early-Onset Infection With SARS-CoV-2 in 33 Neonates Born to Mothers With COVID-19 in Wuhan, China. JAMA Pediatr. *174,* 722-725.

Zhou, P., Yang, X.L., Wang, X.G., et al. (2020). A pneumonia outbreak associated with a new coronavirus of probable bat origin. Nature *579*, 270-273.

**Table 1 The congenital transmission of SARS-CoV-2.**

| Placental investigation | Virus in amniotic fluid | Testing methods | Time points of detection in newborns | Age of infected mother (weeks of gestation) | Reference |
|---|---|---|---|---|---|
| Viral load, signs of acute and chronic intervillous inflammation | Positive | Positive reverse transcription–polymerase chain reaction (RT–PCR) result in placenta, neonatal specimens, and amniotic fluid; detection of SARS-CoV-2 N protein by antibody test in perivillous trophoblastic cells | Nasopharyngeal and rectal swabs collected at 1 h and on PND 13 and PND 18 | 23-year-old (35 weeks) | Vivanti et al. (2020) |
| Virions invading syncytiotrophoblasts in placental villi by electron microscopy analysis | NA | Neonate negative for RT–PCR test on PND 2 and PND 3 | Nasopharyngeal swabs obtained on PND 2 and PND 3 | 40-year-old (28 weeks) | Algarroba et al. (2020) |
| NA | NA | Neonate positive for RT–PCR test | Nasopharyngeal swab at 16 h after delivery | 41-year-old (33 weeks) | Alzamora et al. (2020) |
| NA | NA | Neonate negative for RT–PCR test; positive for SARS-CoV-2 IgM and IgG; elevated cytokine levels (IL-6 and IL-10) | Neonatal samples obtained at 2 h of age for SARS-CoV-2 IgM and IgG antibody tests; nasopharyngeal swabs taken from 2 h to PND 16 for five RT–PCR tests | 29-year-old (34 weeks) | Dong et al. (2020) |
| NA | Positive | RT–PCR test positive in the infant | Negative for nasal and throat swabs obtained after delivery, but positive 24 h later | 22-year-old (32 weeks) | Zamaniyan et al. (2020) |
| Positive for viral RNA | NA | Positive RT–PCR result in placenta and neonatal specimen | Nasopharygeal swab collected at birth | Two cases (34 and 39 weeks, respectively) | Fenizia et al. (2020) |

**Figure legends**

**Figure 1** Schematic illustration of the replication cycle of SARS-CoV-2. The entry of SARS-CoV-2 into host cells starts with the binding of S proteins with the receptor ACE2. The host cell surface protease TMPRSS2 activates S protein, which triggers fusion of the viral and host plasma membranes. SARS-CoV-2 can also enter the cell via endocytosis. In this pathway, the fusion of viral and lysosomal membranes is triggered by cathepsins. The autoproteolytic cleavage of viral polyproteins pp1a and pp1ab generates 16 Nsps with various functions. The RTCs anchor on DMVs, which appear to originate from the ER. The viral RNAs are stored in DMVs and transported to the cytosol for translation or viral assembly via double-membrane-spanning pores. Viral particles are assembled in the ER and/or ERGIC. The virions traffic to late endosomes/lysosomes and egress via the lysosomal exocytosis pathway.

http://mc.manuscriptcentral.com/jmcb

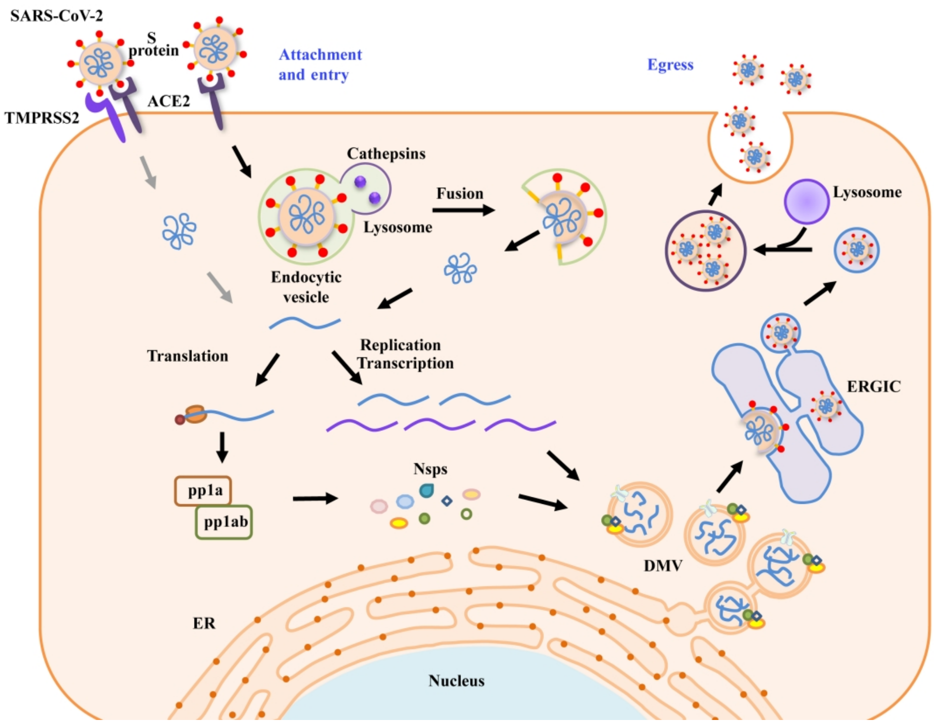

Figure 1. Schematic illustration of the replication cycle of SARS-CoV-2.

254x190mm (300 x 300 DPI)
